# Multisystem Complications in Spinal Muscular Atrophy Type III: Chronic Respiratory Failure and Intractable Epilepsy

**DOI:** 10.7759/cureus.108909

**Published:** 2026-05-15

**Authors:** Olivia R Kaufman, Christopher M Ahmad, Samira Haberman, Curtis King

**Affiliations:** 1 Department of Medicine, Kansas City University of Medicine and Biosciences, Joplin, USA; 2 Department of Primary Care, Kansas City University, Joplin, USA

**Keywords:** chronic respiratory failure, clobazam, felbamate, generalized epilepsy, hypercapnia, kugelberg-welander disease, non-invasive ventilation, scoliosis, sma type iii, spinal muscular atrophy

## Abstract

Spinal muscular atrophy (SMA) is a rare neuromuscular disorder characterized by progressive motor weakness with multisystem involvement. Type III SMA, also known as Kugelberg-Welander disease, typically presents in childhood and allows survival into adolescence and adulthood, during which cumulative complications may emerge. We report the case of a 19-year-old female patient with genetically confirmed SMA type III (zero copies of SMN1, two copies of SMN2) whose disease course was complicated by chronic respiratory failure with hypercapnia requiring non-invasive ventilation and intractable generalized epilepsy. She was born prematurely at seven and a half months via emergent cesarean section for placenta previa and maternal hemorrhage, requiring a one-month neonatal intensive care unit stay with supplemental oxygen. SMA was diagnosed at 28 months of age following early motor weakness. Over childhood, she developed choking with thin liquids, weak cough during viral illnesses, and recurrent respiratory infections, with progression to restrictive lung disease and impaired airway clearance. At age 11, she developed head-drop seizures and was diagnosed with intractable generalized idiopathic epilepsy. Orthopedic complications included neuromuscular scoliosis requiring spinal fusion. This case underscores how SMA type III evolves into a multisystem disorder with cumulative respiratory, neurologic, and orthopedic morbidity, highlighting the need for vigilant longitudinal surveillance, proactive respiratory support, individualized seizure management, and coordinated interdisciplinary care.

## Introduction

Spinal muscular atrophy (SMA) type III, or Kugelberg-Welander disease, is an uncommon childhood-onset neuromuscular condition caused by loss of SMN1 function with relative preservation through SMN2 copy number [1]. Unlike more severe SMA phenotypes, individuals with type III often achieve early motor milestones and survive into adolescence and adulthood. However, long-term disease trajectories may include progressive respiratory compromise, musculoskeletal deformities, and additional neurologic comorbidities [2,3].

SMA type III demonstrates substantial clinical heterogeneity, with patients exhibiting variable rates of motor decline and systemic complications over time. Although historically categorized primarily as a motor neuron disorder, emerging literature suggests that SMA may involve broader systemic dysfunction affecting respiratory physiology, musculoskeletal development, and metabolic regulation. This evolving understanding has prompted increasing emphasis on longitudinal surveillance strategies that extend beyond neuromuscular assessment alone, particularly as advances in therapies have improved survival and functional outcomes in this population [4].

As survival improves and patients age, clinicians increasingly encounter non-motor complications that dominate morbidity and healthcare utilization. This report describes a young adult with SMA type III whose course was marked by chronic respiratory failure with hypercapnia, dependence on non-invasive ventilation, refractory epilepsy, and orthopedic sequelae. The purpose of this case is to illustrate the cumulative, multisystem nature of SMA type III and emphasize the importance of anticipatory, interdisciplinary management across the lifespan [4-6].

While respiratory decline and musculoskeletal complications are well-characterized in SMA type III, the coexistence of refractory epilepsy remains poorly described and mechanistically unclear. Although SMA is traditionally considered a lower motor neuron disorder, emerging evidence suggests broader neurologic involvement in select phenotypes, raising questions about central nervous system vulnerability and potential genotype-phenotype variability.

This case is notable for the early onset of intractable generalized epilepsy in the setting of genetically confirmed SMA type III, in addition to progressive respiratory failure requiring non-invasive ventilation. By presenting this longitudinal trajectory, we aim to highlight a less commonly emphasized neurologic comorbidity, contextualize it within the patient’s multisystem disease burden, and underscore the importance of anticipating atypical manifestations in long-term SMA care.

## Case presentation

The patient is a 19-year-old female with a known diagnosis of SMA type III. She was born prematurely at seven and a half months’ gestation, weighing three pounds nine ounces, following an emergent cesarean section for placenta previa complicated by maternal hemorrhage. Her neonatal course required a four-week stay in the neonatal intensive care unit, including approximately two weeks of supplemental oxygen, without the need for intubation.

**Figure 1:**
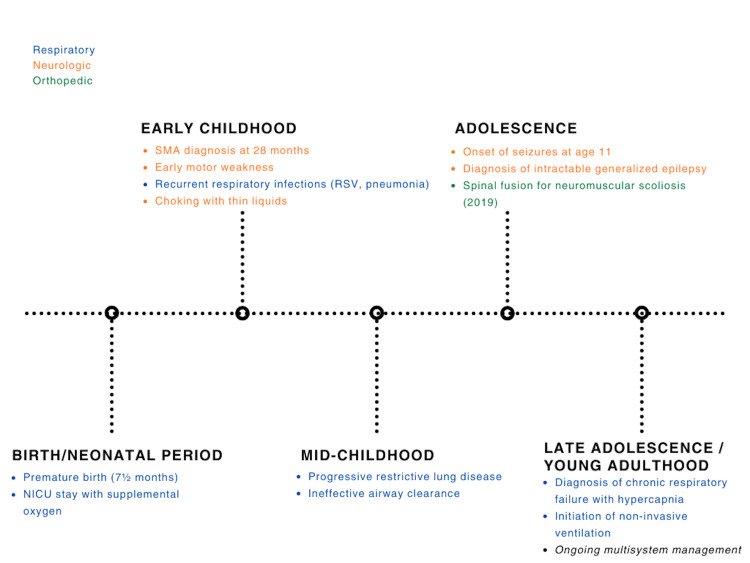
Timeline illustrating the progression of spinal muscular atrophy type III from early diagnosis through the development of chronic respiratory failure, epilepsy, and orthopedic complications, highlighting cumulative multisystem burden over time. Figure created by Olivia Kaufman using Canva

Although weakness was noted earlier in life, formal diagnosis of SMA occurred at 28 months of age. The patient’s clinical trajectory, from early motor symptoms through the development of respiratory, neurologic, and orthopedic complications, is summarized in Figure 1.

Genetic testing confirmed zero copies of SMN1 and two copies of SMN2. During early childhood, she experienced choking with thin liquids, a weak cough during viral illnesses, respiratory syncytial virus infection at one year of age, and pneumonia at two years of age, both managed without hospitalization.

Over time, she developed restrictive lung disease with ineffective airway clearance, clinically characterized by progressive ventilatory insufficiency and hypercapnia. Formal pulmonary function testing data were not consistently available across all timepoints; however, the clinical trajectory was marked by declining respiratory reserve and eventual need for non-invasive ventilatory support.

Neurologic complications emerged at age 11, when she began experiencing head-drop seizures. She was diagnosed with intractable generalized idiopathic epilepsy without status epilepticus. Her seizure management regimen included clobazam and felbamate, with intranasal midazolam (Nayzilam) prescribed as rescue therapy. Despite multidrug therapy, seizures persisted. Seizure semiology was characterized by abrupt head-drop episodes without clear focal onset, raising concern for generalized atonic or myoclonic features. Seizure frequency persisted despite multidrug therapy, meeting criteria for intractable epilepsy, defined by persistence despite multiple appropriately dosed antiepileptic medications. Electroencephalographic data were not consistently available for longitudinal comparison, representing a limitation in further subclassification.

**Figure 2:**
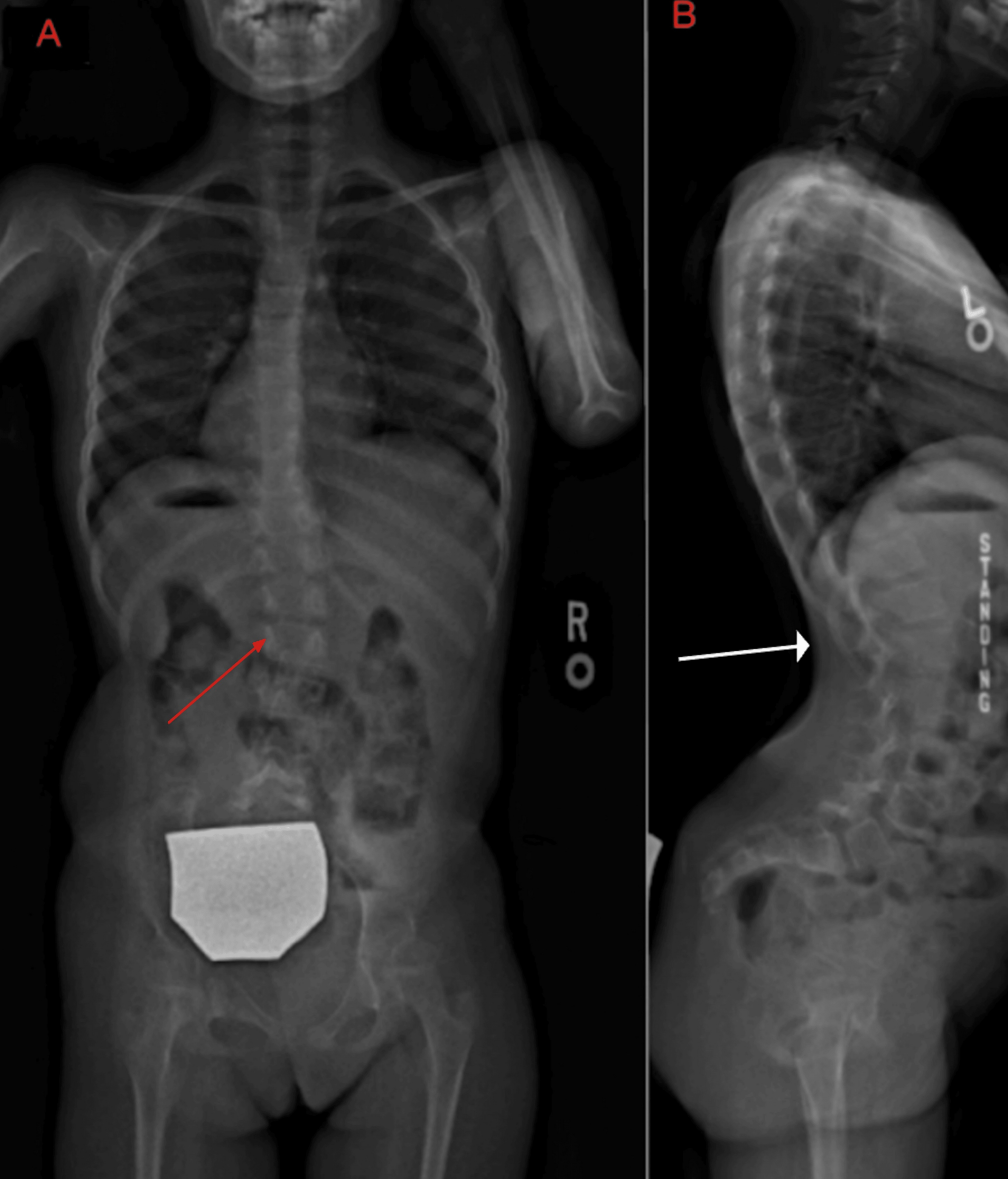
Radiographic evaluation of neuromuscular scoliosis in spinal muscular atrophy type III. (A) Standing anteroposterior radiograph demonstrating thoracolumbar scoliosis (red arrow). (B) Lateral radiograph illustrating sagittal spinal deformity affecting thoracic alignment (white arrow). These findings preceded surgical correction with spinal fusion. A radiopaque pelvic density is present and represents external shielding artifact rather than underlying pathology.

Orthopedic manifestations included neuromuscular scoliosis of the thoracolumbar spine, for which she underwent spinal fusion in 2019 as shown in Figure 2. Additional documented conditions included maxillary and mandibular hypoplasia, intellectual disability, and generalized anxiety disorder. These were considered within the broader context of chronic disease burden, though a direct causal relationship with SMA could not be definitively established.

**Figure 3:**
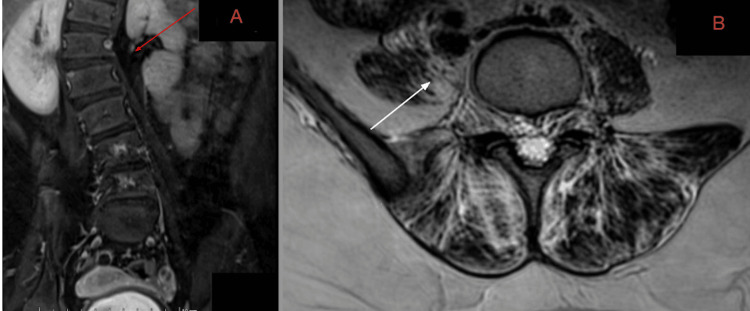
MRI findings in spinal muscular atrophy type III. (A) Coronal MRI demonstrating thoracolumbar spinal curvature (red arrow). (B) Axial MRI demonstrating fatty infiltration and atrophy of paraspinal musculature (white arrow) consistent with neuromuscular disease. MRI: magnetic resonance imaging

MRI of the thoracolumbar spine demonstrated scoliosis with associated paraspinal muscle atrophy and fatty infiltration consistent with chronic neuromuscular degeneration (Figure 3).

At the time of reporting, her active medications included clobazam 20 mg twice daily, felbamate 600 mg twice daily (morning and noon) and 400 mg nightly, risdiplam (Evrysdi), tolterodine for bladder dysfunction, and intranasal midazolam as needed for seizure rescue. Supportive therapies included acetaminophen, ibuprofen, vitamin D, multivitamins, lactobacillus probiotic, inulin, and polyethylene glycol. The patient’s multisystem complications, associated interventions, and ongoing management needs are summarized in Table 1.

**Table 1:** Summary of major clinical complications and interventions. SMA: Spinal muscular atrophy

System	Key Manifestations	Interventions	Ongoing Needs
Respiratory	Restrictive lung disease, hypercapnia	Non-invasive ventilation	Pulmonary monitoring, airway clearance
Neurologic	Intractable generalized epilepsy	Clobazam, felbamate, intranasal midazolam	Seizure surveillance, medication adjustment
Orthopedic	Neuromuscular scoliosis	Spinal fusion	Long-term orthopedic follow-up
Genetic/Disease-modifying	SMA type III	Risdiplam (Evrysdi)	Ongoing disease-modifying therapy
Supportive	Anxiety, intellectual disability	Multidisciplinary support	Psychosocial care

## Discussion

**Figure 4:**
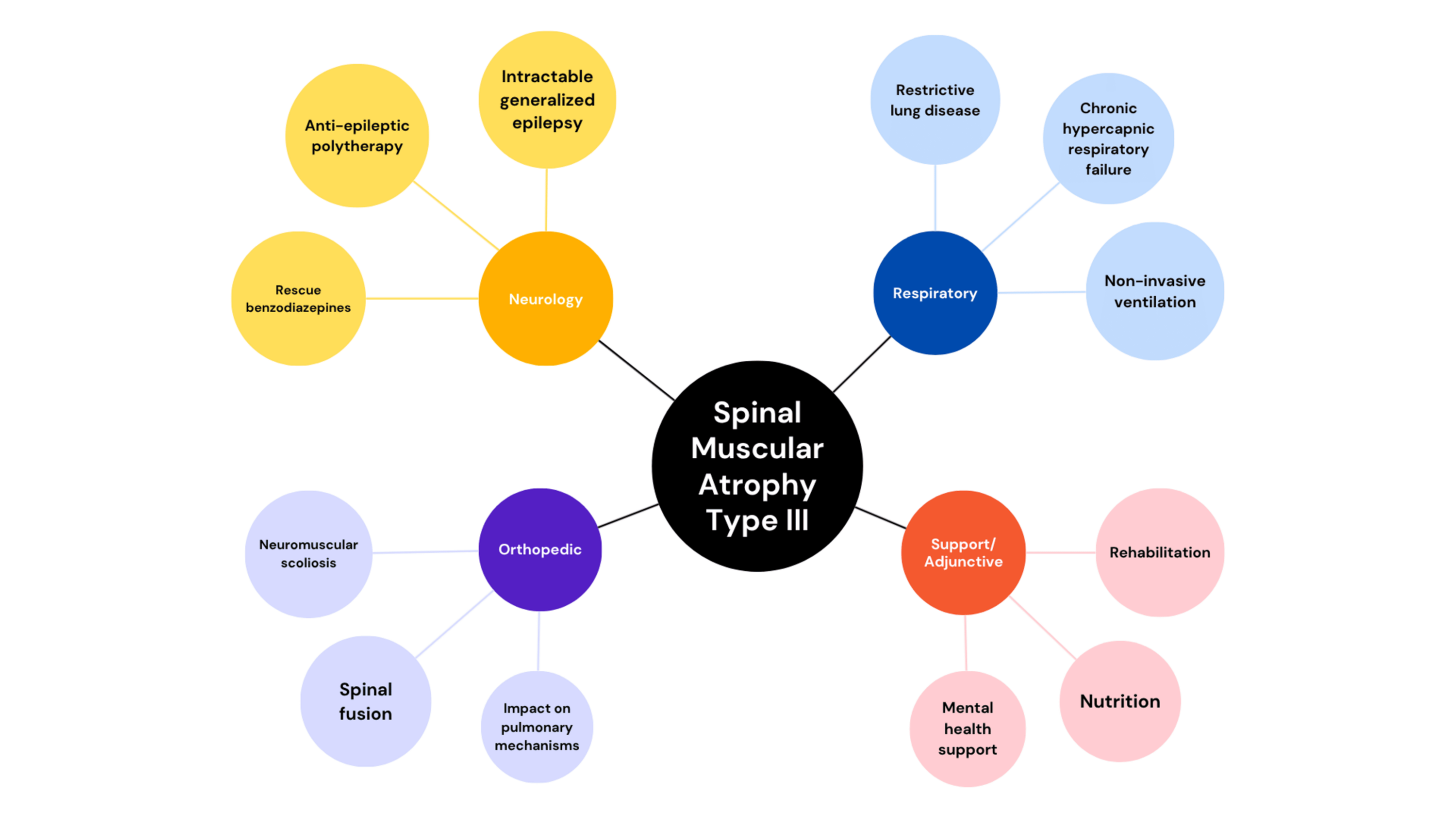
Conceptual map illustrating the interrelated respiratory, neurologic, and orthopedic complications of SMA type III and the necessity of coordinated interdisciplinary management. SMA: Spinal muscular atrophy Figure created by Olivia Kaufman using Canva

This case illustrates the cumulative multisystem complexity of SMA type III as complications accrue over time (Figure 4). While SMA type III is primarily characterized by progressive motor weakness and delayed loss of ambulation, this patient’s trajectory underscores the importance of recognizing and anticipating respiratory, neurologic, and orthopedic manifestations that extend well beyond motor decline [7].

Respiratory compromise is a well-established driver of morbidity in SMA, and this case highlights how subtle early features may foreshadow long-term ventilatory dependence. Early signs of respiratory vulnerability such as ineffective airway clearance, choking with thin liquids, and recurrent childhood infections, preceded the later development of restrictive lung disease and chronic respiratory failure with hypercapnia [8,9]. Progressive weakness of the diaphragm and intercostal musculature contributes to restrictive ventilatory physiology, diminished tidal volumes, and impaired nocturnal ventilation. These can lead to chronic carbon dioxide retention and sleep-disordered breathing, ultimately necessitating ventilatory support [9]. The progression from mild respiratory symptoms to dependence on non-invasive ventilation emphasizes the need for proactive pulmonary surveillance and timely intervention in patients with SMA type III [10]. This progression is consistent with the expected trajectory of progressive ventilatory compromise observed clinically in patients with SMA type II and III, even in the setting of disease-modifying therapy. While the patient was receiving disease-modifying therapy with risdiplam, the absence of standardized longitudinal functional data limited the ability to assess its impact on disease progression in this case.

The emergence of seizures at age 11 represents a less typical but clinically significant component of this patient’s disease course. Epilepsy is not classically associated with SMA type III, and its presence raises important considerations regarding potential phenotypic variability, coincidental comorbidity, or broader neurologic involvement beyond lower motor neuron pathology [11,12]. In this patient, intractable generalized epilepsy required multidrug antiepileptic therapy and intranasal rescue medication, complicating daily management and increasing the risk of treatment-related adverse effects. This underscores the importance of maintaining vigilance for atypical neurologic manifestations in SMA and individualizing antiepileptic regimens in the context of underlying neuromuscular disease. Although a definitive mechanistic link remains unclear, the coexistence of these conditions raises consideration of broader neurologic involvement beyond traditional lower motor neuron pathology.

Neuromuscular scoliosis requiring spinal fusion further compounded morbidity, with direct implications for pulmonary mechanics and overall quality of life. Orthopedic complications are frequent in SMA and often necessitate surgical intervention [8,13,14]. In this patient, spinal fusion represented not only a structural correction but also a critical intersection between orthopedic, respiratory, and neurologic care, highlighting the interdependence of these systems in neuromuscular disease management.

Management of SMA increasingly relies on coordinated multidisciplinary care models that integrate pulmonology, neurology, orthopedics, rehabilitation medicine, and psychosocial support. Structured multidisciplinary clinics have been shown to improve care coordination, facilitate earlier recognition of complications, and enhance quality of life for patients living with complex neuromuscular conditions [15].

Taken together, this case illustrates the cumulative nature of SMA type III care (Figure 4). Respiratory failure, refractory epilepsy, and orthopedic deformities amplify one another, reinforcing the necessity of coordinated interdisciplinary management involving pulmonology, neurology, orthopedics, rehabilitation, and primary care [8]. As patients with SMA increasingly survive into adolescence and adulthood, anticipatory, integrated care models become essential to optimizing long-term outcomes.

This case describes a single patient, which limits generalizability to the broader population of individuals with SMA type III. The retrospective nature of the review introduces potential gaps in longitudinal data, including incomplete standardized pulmonary function trends, detailed seizure frequency tracking, and validated quality-of-life assessments across developmental stages. Additionally, the patient’s history of prematurity and multiple comorbid conditions makes it difficult to fully delineate the independent contribution of SMA to each complication observed. While disease-modifying therapy was initiated, the absence of structured pre- and post-treatment functional comparisons limits assessment of its impact on respiratory or neurologic progression. As with all descriptive case reports, the findings are hypothesis-generating rather than definitive, and long-term adult outcomes remain under continued follow-up.

## Conclusions

This case emphasizes that SMA type III extends beyond motor weakness to encompass chronic respiratory failure, refractory epilepsy, and orthopedic deformities. As patients with SMA increasingly survive into adolescence and adulthood, clinicians must anticipate multisystem complications and adopt an integrative, interdisciplinary approach to care. This case contributes to the evolving understanding of SMA type III by illustrating a rare coexistence of chronic respiratory failure and refractory epilepsy, emphasizing the need for clinicians to remain vigilant for atypical neurologic comorbidities in addition to well-recognized respiratory and orthopedic complications.
